# A New Quaternion-Based Kalman Filter for Real-Time Attitude Estimation Using the Two-Step Geometrically-Intuitive Correction Algorithm

**DOI:** 10.3390/s17092146

**Published:** 2017-09-19

**Authors:** Kaiqiang Feng, Jie Li, Xiaoming Zhang, Chong Shen, Yu Bi, Tao Zheng, Jun Liu

**Affiliations:** 1 Key Laboratory of instrumentation Science & Dynamic Measurement, Ministry of Education, North University of China, Taiyuan 030051, China; b1506011@st.nuc.edu.cn (K.F.); zxm_auto@nuc.edu.cn (X.Z.); shenchong@nuc.edu.cn (C.S.); b1506009@st.nuc.edu.cn (Y.B.); s1506044@st.nuc.edu.cn (T.Z.); liuj@nuc.edu.cn (J.L.); 2 National Key Laboratory for Electronic Measurement Technology, North University of China, Taiyuan 030051, China

**Keywords:** AHRS, attitude estimation, magnetic distortion, two-step geometrically intuitive correction (TGIC), Kalman filter

## Abstract

In order to reduce the computational complexity, and improve the pitch/roll estimation accuracy of the low-cost attitude heading reference system (AHRS) under conditions of magnetic-distortion, a novel linear Kalman filter, suitable for nonlinear attitude estimation, is proposed in this paper. The new algorithm is the combination of two-step geometrically-intuitive correction (TGIC) and the Kalman filter. In the proposed algorithm, the sequential two-step geometrically-intuitive correction scheme is used to make the current estimation of pitch/roll immune to magnetic distortion. Meanwhile, the TGIC produces a computed quaternion input for the Kalman filter, which avoids the linearization error of measurement equations and reduces the computational complexity. Several experiments have been carried out to validate the performance of the filter design. The results demonstrate that the mean time consumption and the root mean square error (RMSE) of pitch/roll estimation under magnetic disturbances are reduced by 45.9% and 33.8%, respectively, when compared with a standard filter. In addition, the proposed filter is applicable for attitude estimation under various dynamic conditions.

## 1. Introduction

Accurate orientation estimation is essential for navigation in a wide range of applications, such as unmanned aerial vehicle (UAV) navigation, mobile devices [1,2], autonomous underwater vehicle (AUV) navigation and human body motion tracking, etc. [3,4,5], in the industrial and military fields. A strap-down MARG (magnetic, angular rate, and gravity) system, also known as AHRS (attitude and heading reference system) [6,7,8,9] is commonly used to determine the orientation of a moving object in three-dimensional spaces. Theoretically, the AHRS can determine the 3-D orientation with gravity and magnetic field measurements from the accelerometer and magnetometer, or propagates the attitude by integrating gyroscope output from known initial conditions. However, due to inertial and magnetic sensors having their own disadvantages, a single type sensor is unable to provide precise attitude information. For example, an accelerometer measures not only the gravitational direction but also the linear acceleration of the vehicle in dynamic situations. In this case, it is difficult to dissociate the linear acceleration from the gravity and calculate the attitude accurately. A gyroscope, especially the low-grade Micro-Electro-Mechanical System (MEMS) sensor, is vulnerable to low-frequency drift and wideband measurement noise, resulting in boundless orientation drift errors, as the measurement errors are accumulated when the data is integrated. The reading from the magnetometers is easily influenced by ferrous material in the vicinity of the sensor. Therefore, it needs to fuse data coming from separate sensors to provide an optimal estimate of orientation.

In the last decades, lots of orientation estimation algorithms fusing inertial and magnetic sensors have been performed and the most commonly used approaches are the complementary filter [10,11,12,13,14] and extend Kalman filter (EKF). In [10], Mahony et al. proposed an explicit complementary filter (ECF) for the orientation estimation of UAV. Such a filter utilizes a proportional-integral (PI) controller to estimate the gyro biases on-line and provide good attitude estimates. Madgwick et al. present a computationally efficient gradient descent algorithm for use in a human motion tracking system given measurement from MARG sensor arrays in [12]. The proposed algorithm can produce better performance at a lower computational cost and is able to reduce the effect of the magnetic disturbance. In both the complementary filter and gradient descent algorithm, the data from the gyroscope is integrated to obtain orientation, while the data from the accelerometer and magnetometer are used to estimate the gyroscope biases online. However, it should be noted that they all belong to the constant gain complementary filter and the estimation accuracy of the two methods depends on both the accelerometer and magnetometer.

The Kalman filter (KF) and extend Kalman filter (EKF), as the most well-known and widely adopted approaches, have been applied in diverse areas, especially in orientation estimation [15,16]. Sabatini et al. [17] present a quaternion-based extend Kalman filter for human body tracking. In the proposed method, the quaternion associated with the bias of the accelerometer and magnetometer is modeled as the state vector to estimate the bias of the gyroscope for online calibration. Moreover, the author presents an adaptive mechanism to guard against the non-gravitation and magnetic disturbance. Trawny et al. [18] develop an indirect extend Kalman filter (EKF) where the error is defined as a small angle rotation between the true and estimate vector. In this approach, a three-dimension error angle vector together with the three-dimension bias of a gyroscope are used as the state vector, which reduces the seven-dimension of the state vector in the traditional EKF and improves the stability of the filter. However, it should be noted that the implementation of EKF would induce a linearization error in the Kalman filter and increase the computational complexity.

To avoid the linearization procedure of the measurement model and reduce the computational loads of the quaternion-based EKF, a two-layer filter architecture has been presented in [19,20,21]. The first layer is to obtain the observation quaternion by preprocessing the accelerometer and magnetometer measurements using an external quaternion estimator (QUEST) algorithm. The second layer is the line Kalman filter that utilizes 4-D quaternions as the state vector and the outputs of the first layer as the observation vector, which avoids the linearization of the observation model and results in a simplification of the Kalman filter design. In this two-layer strategy, the main difference of the researchers’ work is to use a different external quaternion estimator to produce observation quaternions. In [22], Marins et al. describe a Gauss-Newton algorithm (GNA) designed for computing the quaternion input for a Kalman filter. Liu et al. [23] simplified this approach by obtaining the optimal weights of each measurement by analyzing the error variance. In order to reduce the computational complexity further and enhance the system’s dynamic tracking characteristics, Wang et al. present an adaptive-step gradient descent algorithm (ASGD) in [21] recently. Although an iterative method such as the Gauss-Newton algorithm (GNA) and gradient descent algorithm (GDA) can produce the computed quaternion, they consume too much time and space. Similarity to the work described above, Yun et al. describe the QUEST and factored quaternion algorithm (FQA) in [20] and [24] respectively. The QUEST [25] and FQA are both solutions to Wahba’s problem [26,27] and the main difference between them is the computation speed. The QUEST algorithm finds the best-fit attitude quaternion by minimizing a loss function. The FQA provides a more efficient deterministic solution for the attitude based on gravity and magnetic field vectors and can obtain the accuracy that is identical to that of QUEST algorithm. However, it can be seen from [20,28] that the estimate from single-frame algorithms like QUEST and FQA could produce large errors when the system is in dynamic conditions. 

This paper describes the design and implementation of the quaternion-based line Kalman filter for AHRS using the two-layer filter architecture described above. Unlike the state-of-the-art external QUEST approach, the presented algorithm provides the computed quaternion by using a two-step correction sequence. The first step correction regains the pitch/roll information by aligning the estimated direction of gravity with the upward direction, while the second step correction revises the yaw angle by pointing the estimated direction of the local magnetic field to north. The decoupling of accelerometer and magnetometer measurement eliminates the influence of magnetic distortion on the determination of pitch/roll. In addition, a magnetic field detection and step-skip scheme is proposed to guard against the magnetic distortion on the estimation of the yaw angle. 

The structure of the paper is as follows. Section 2 briefly sets out knowledge regarding accelerometer/magnetometer attitude determination and presents details of the proposed quaternion-based attitude estimation scheme. The experiment results are presented and discussed in Section 3. Section 4 is the conclusion.

## 2. Methods

### 2.1. Orientation Representation and Determination

#### 2.1.1. Quaternion-Based Orientation Representation

In order to describe the orientation, we define the body frame *b*{*xyz*} and the navigation frame n{North, Up, East (NUE)}. The *x*-axis is aligned with the forward direction, the *y*-axis points to the top of the body and the *z*-axis refers to the right direction.

The orientation of the rigid body can be derived from an attitude transformation matrix $C_{n}^{b}$. $C_{n}^{b}$ is an orthogonal matrix and can be carried out through three different separate rotations about the three axes. The first rotation is about the *y*-axis by *ψ*, the second rotation is about the z-axis by *θ*, and the third rotation is about the x-axis by *γ*; they are defined as:

(1) $$C_{\psi }^{y}=\left[\begin{array}{ccc} \cos\psi & 0 & \sin\psi \\ 0 & 1 & 0\\ -\sin\psi & 0 & \cos\psi \end{array}\right],C_{\theta }^{z}=\left[\begin{array}{ccc} \cos\theta & -\sin\theta & 0\\ \sin\theta & \cos\theta & 0\\ 0 & 0 & 1 \end{array}\right],C_{\gamma }^{x}=\left[\begin{array}{ccc} 1 & 0 & 0\\ 0 & \cos\gamma & -\sin\gamma \\ 0 & \sin\gamma & \cos\gamma \end{array}\right]$$

Then:

(2) $$C_{n}^{b}=C_{\gamma }^{x}C_{\theta }^{z}C_{\psi }^{y}=\left[\begin{array}{ccc} \cos\theta \cos\psi & \sin\theta & -\sin\psi \cos\theta \\ -\cos\gamma \cos\varphi \sin\theta +\sin\gamma \sin\psi & \cos\gamma \cos\theta & \sin\psi \sin\theta \cos\gamma +\cos\psi \sin\gamma \\ \sin\theta \sin\gamma \cos\psi +\cos\gamma \sin\psi & -\sin\gamma \cos\theta & -\sin\theta \sin\gamma \sin\psi +\cos\psi \cos\gamma \end{array}\right]$$

Due to the drawbacks of the Euler angle representation, the quaternion qnb=[q0q1q2q3] is used to represent the attitude of the n frame in respect to the b frame and the equivalent rotations from the n frame to the *b* frame can be expressed using the following equation:

(3) pb=qnb⊗pn⊗q∗nb=M(qnb)M′(qnb∗)pn

where the symbol ⊗ indicates the quaternion multiplication; $p^{b}$ and $p^{n}$ describe the observation vector p expressed in the b frame and n frame, respectively; and q∗nb is the conjugate quaternion of qnb and can be expressed as:

(4) q∗nb=[q0−q1−q2−q3]

Thus, the direction cosine matrix (DCM) can be rewritten in quaternion form as

(5) $$C_{n}^{b}=\left[\begin{array}{ccc} q0^{2}+q1^{2}-q2^{2}-q3^{2} & 2q0q3+2q1q2 & -2q0q2+2q1q3\\ -2q0q3+2q1q2 & q0^{2}-q1^{2}+q2^{2}-q3^{2} & 2q0q1+2q2q3\\ 2q0q2+2q1q3 & -2q0q1+2q2q3 & q0^{2}-q1^{2}-q2^{2}+q3^{2} \end{array}\right]$$

According to [29], the attitude angles ψ, θ and γ can be calculated as:

(6) $$\left\{ \begin{array}{l} \psi =ac\tan((2q0q2-2q1q3)/(q0^{2}+q1^{2}-q2^{2}-q3^{2}))\\ \theta =ac\sin(2q0q3+2q1q2)\\ \gamma =ac\tan((2q0q1-2q2q3)/(q0^{2}-q1^{2}+q2^{2}-q3^{2})) \end{array}\right.$$

#### 2.1.2. Accelerometer/Magnetometer-Based Attitude Determination

The accelerometer can determine the pitch and roll of the body by measuring the gravitational acceleration during static or quasi-static conditions; the magnetometer can determine the direction of the body by measuring the geomagnetic field based on the pitch and roll information provided by the accelerometer under the condition of no magnetic disturbance, then the whole attitude information of the body can be obtained.

A. Pitch and Roll Determination from Accelerometer

When the vehicle is stationary, the measurement of gravitational acceleration in the body frame can be expressed as:

(7) $$\left[\begin{array}{c} f_{x}\\ f_{y}\\ f_{z} \end{array}\right]=C_{n}^{b}\left[\begin{array}{c} 0\\ g\\ 0 \end{array}\right]=\left[\begin{array}{c} g\sin\theta \\ g\cos\theta \sin\gamma \\ -g\cos\theta \cos\gamma \end{array}\right]$$

where $f_{x}$, $f_{y}$ and $f_{z}$ denote the measurements of the accelerometer in the body frame; and g represents the local gravitational acceleration.

Then, the pitch and roll can be obtained:

(8) θ=acsin(fxg)

(9) γ=actan(−fyfz)

B. Heading Determination from the Magnetometer

Since the accelerometer can be only used to measure the angles relative to the horizontal plane, in order to obtain the heading of the vehicle, the tri-axial magnetometer is utilized to determine the direction of geomagnetic north by measuring the local magnetic field. Assuming that the component of the earth’s magnetic field vector in the body frame is $H^{b}={\left[\begin{array}{ccc} H_{x}^{b} & H_{y}^{b} & H_{z}^{b} \end{array}\right]}^{T}$, the horizontal component of the earth’s magnetic field vector $H^{l}={\left[\begin{array}{ccc} H_{x}^{l} & H_{y}^{l} & H_{z}^{l} \end{array}\right]}^{T}$ can be calculated by:

(10) $$\left[\begin{array}{c} H_{x}^{l}\\ H_{y}^{l}\\ H_{z}^{l} \end{array}\right]=\left[\begin{array}{ccc} \cos\theta & \sin\theta & 0\\ -\cos\varphi \sin\theta & \cos\gamma \cos\theta & \sin\gamma \\ \sin\theta \sin\gamma & -\sin\gamma \cos\theta & \cos\gamma \end{array}\right]\left[\begin{array}{c} H_{x}^{b}\\ H_{y}^{b}\\ H_{z}^{b} \end{array}\right]$$

where ${\left[\begin{array}{ccc} H_{x}^{b} & H_{y}^{b} & H_{z}^{b} \end{array}\right]}^{T}$ are given by the magnetometer measurements; and the pitch (θ) and the roll (γ) are provided by the accelerometers. Then the heading (ψ) of the vehicle can be defined as:

(11) ψ=atan(HzlHxl)+D

where D represents the magnetic declination, which is the angle between the magnetic north and the geodetic north. This varies and depends on the location of the AHRS.

### 2.2. Data Fusion Based on a Kalman Filter

**Figure 1:**
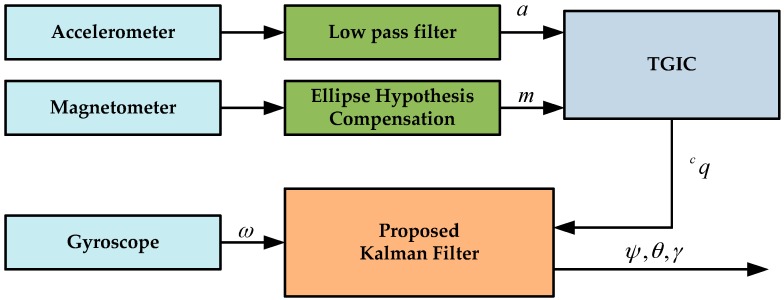
Block diagram of the proposed KF design.

A novel data fusion method based on a Kalman filter will be described in this section. Figure 1 shows the block diagram of the filtering process. It can be seen that the measurements of the accelerometer and magnetometer are used as the input of the two-step geometrically intuitive correction (TGIC) block to produce the computed quaternion, then the computed quaternion is used as the measurement of the line Kalman filter to correct the predicted state obtained by using the output of the gyroscope.

#### 2.2.1. Process Model

In this paper, we choose the quaternion as the state vector, which is different from the 7D vectors in the traditional EKF that is composed of four quaternion components and three gyroscope bias components. The 4D state vectors of the proposed filter can be expressed as $X_{k}={\left[\begin{array}{cccc} q0 & q1 & q2 & q3 \end{array}\right]}^{T}$, and the state equation is described by the following well-known equation [30]:

(12) $$\dot{q}=\Omega (\omega )q$$

where

(13) $$\Omega (\omega )=\frac{1}{2}\left[\begin{array}{cc} 0 & -\omega^{T}\\ \omega & \left[\omega \times \right] \end{array}\right]$$

The term $\omega ={\left[\begin{array}{ccc} \omega_{x} & \omega_{y} & \omega_{z} \end{array}\right]}^{T}$ is the angular rate for the *x*, *y* and *z* axis in sensor frame; and [ω×] denotes the skew symmetric matrix that is associated with ω and is equal to:

(14) $$\left[\omega \times \right]=\left[\begin{array}{ccc} 0 & \omega_{z} & -\omega_{y}\\ -\omega_{z} & 0 & \omega_{x}\\ \omega_{y} & -\omega_{x} & 0 \end{array}\right]$$

According to [18], the discrete-time form of the system process model can be described as:

(15) $$q_{k+1}=\exp\left(\Omega_{k}\Delta T\right)q_{k}+w_{k},k=0,1,2,\ldots$$

where ΔT represents the system sample interval; $w_{k}$ is the process noise and the covariance matrix of this that can be obtained by (34); and $q_{k}$ and $q_{k+1}$ are the quaternions at time kΔT and (k+1)ΔT respectively. When ΔT is small enough, $\Omega_{k}$ is assumed to be constant in the interval [kΔT,(k+1)ΔT]. Therefore, we can rewrite $\exp\left(\Omega_{k}\Delta T\right)$ using its first-order and second-order items of Taylor series expansion approximately, that is:

(16) $$q_{k+1}=\left(I_{4\times 4}+\frac{1}{2}\Omega_{k}\Delta T\right)q_{k}+w_{k}$$

#### 2.2.2. Observation Model

**Figure 2:**
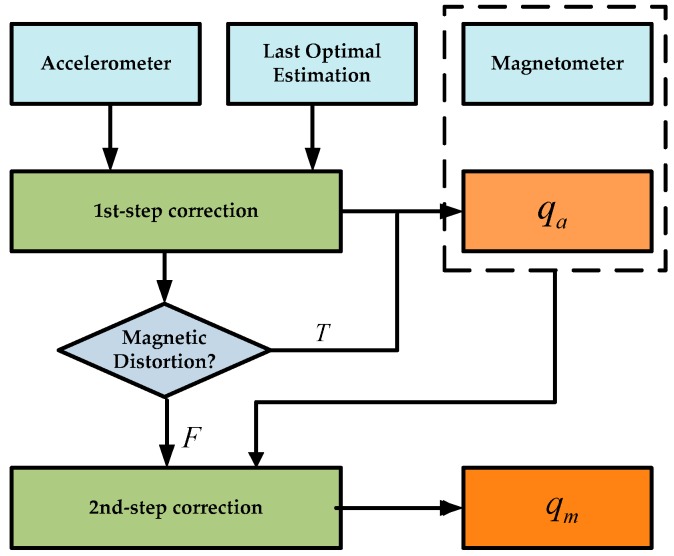
Block diagram of the TGIC.

In this study, the system observation vector is given by:

(17) Zk=[qc0qc1qc2qc3]T

where qc is the computed quaternion produced by the proposed two-step geometrically intuitive correction approach that uses the data from the accelerometer to estimate the gravity direction (upward) and the magnetometer to estimate the direction of magnetic field (northward). Moreover, two correction factors were introduced in the proposed method, which significantly improved the estimation accuracy when the system is in the condition of translational motion and magnetic interference. The implementation of the proposed two-step geometrically intuitive correction approach is depicted in detail in Figure 2.

Firstly, the estimated vector of gravity field and magnetic field are given by:

(18) {v^g=g^b‖g^b‖v^m=m^b‖m^b‖,{g^b=Cnb(qnb)gnm^b=Cnb(qnb)mngn=[010]Tmn=[mNmU0]T

where ${\hat{g}}^{b}$ and ${\hat{m}}^{b}$ denote the estimate vector of the gravity field and magnetic field under the body coordinate system; $g^{n}$ and $m^{n}$ stand for the gravity and magnetic vector in the navigation coordinate system; and Cnb(qnb) denotes the direction cosine matrix (DCM) represented by the quaternion which is obtained from the last optimal estimate.

The measured vector of the gravity field and magnetic field is given by:

(19) $$\left\{ \begin{array}{l} v_{g}=\frac{g^{b}}{\left\|g^{b}\right\|}\\ v_{m}=\frac{m^{b}}{\left\|m^{b}\right\|} \end{array}\right.$$

where $g^{b}={\left[\begin{array}{ccc} a_{x} & a_{y} & a_{z} \end{array}\right]}^{T}$ and $m^{b}={\left[\begin{array}{ccc} m_{x} & m_{y} & m_{z} \end{array}\right]}^{T}$ represent the measurement of the accelerometer and magnetometer in the body coordinate system, respectively.

Theoretically, in static conditions with no magnetic disturbance, the direction of the estimated vector of the gravity field and the magnetic field should be aligned with the measured vector, that is $v_{g}={\hat{v}}_{g}$ and $v_{m}={\hat{v}}_{m}$. However, due to the existence of the random error of the MARG sensors and field disturbance (non-gravity acceleration and magnetic field disturbance), there will be a deviation between the measurement and estimate vectors. In order to correct this deviation, we used the proposed geometrically intuitive method to obtain the optimal quaternion $q_{ma}$ from the accelerometer and magnetometer readings. The two-step correction process is described as follows [31]:

**Step 1 Correct the Estimated Direction of Gravity Using Accelerometer Readings**

**Figure 3:**
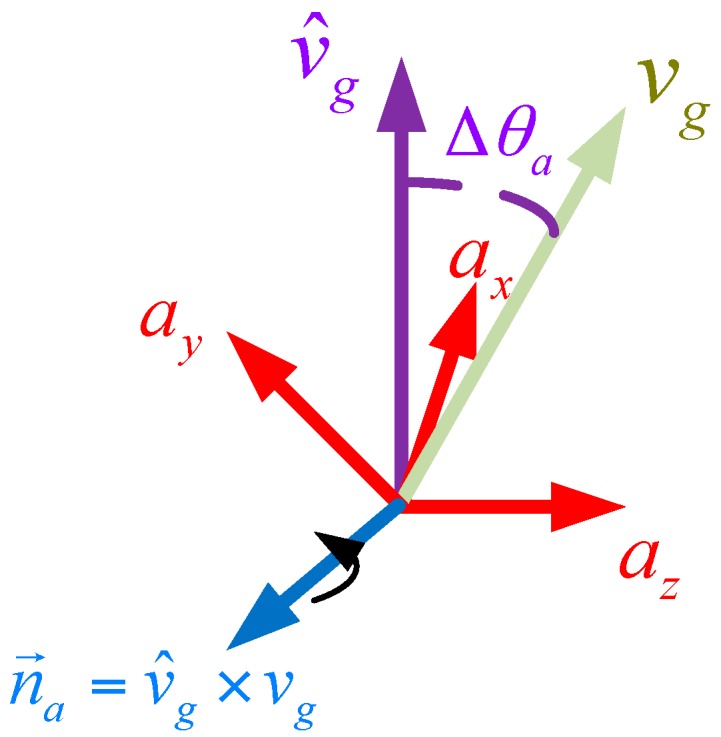
Correcting the estimated direction of gravity using the accelerometer.

As shown in Figure 3, correction for the estimated direction of gravity is performed by rotating the last optimal attitude quaternion $q_{k}$ by the angle $\Delta \theta_{a}$ (the angle between $v_{g}$ and ${\hat{v}}_{g}$) around the axis ${\vec{n}}_{a}$ (which is defined by the cross product of $v_{g}$ and ${\hat{v}}_{g}$). Thus, the corresponding error quaternion $q_{ae}$ and estimated orientation $q_{a}$ can be obtained by:

(20) $$q_{ae}=\cos\left(\frac{\mu_{a}\Delta \theta_{a}}{2}\right)+{\vec{n}}_{a}\sin\left(\frac{\mu_{a}\Delta \theta_{a}}{2}\right)$$

(21) $$q_{a}=q_{ae}⊗q_{k}$$

where ${\vec{n}}_{a}=v_{g}\times {\hat{v}}_{g}$, $\Delta \theta_{a}=a\cos\left(v_{g}\cdot {\hat{v}}_{g}\right)$. The parameter $\mu_{a}$ is used to reduce the influence of the external acceleration. By partially correcting the angle $\Delta \theta_{a}$, the interference of external acceleration will be averaged close to zero. The optimal choice for $\mu_{a}$ is such that the TGIC can obtain a robust attitude in static and dynamic tests without overshooting too much. The determination of the parameter $\mu_{a}$ in various working conditions will be given in the experiment section.

**Step 2 Correct the Estimated Direction of the Magnetic Field Using Magnetometer Readings**

On the basis of the work described in step one, the measured vector of the magnetic field from the magnetometer can be projected onto the horizontal plane by using the quaternion $q_{a}$:

(22) $$v_{mxz}=\left[\begin{array}{cccc} 0 & b_{x} & b_{y} & b_{z} \end{array}\right]=q_{a}⊗\left[\begin{array}{cccc} 0 & m_{x} & m_{y} & m_{z} \end{array}\right]⊗{\left(q_{a}\right)}^{*}$$

Omitting the vertical component of the vector, $v_{mxz}$ can be rewritten as:

(23) $$v_{mxz}=\left[\begin{array}{ccc} \sqrt{b_{x}{}^{2}+b_{z}{}^{2}} & 0 & 0 \end{array}\right]$$

**Figure 4:**
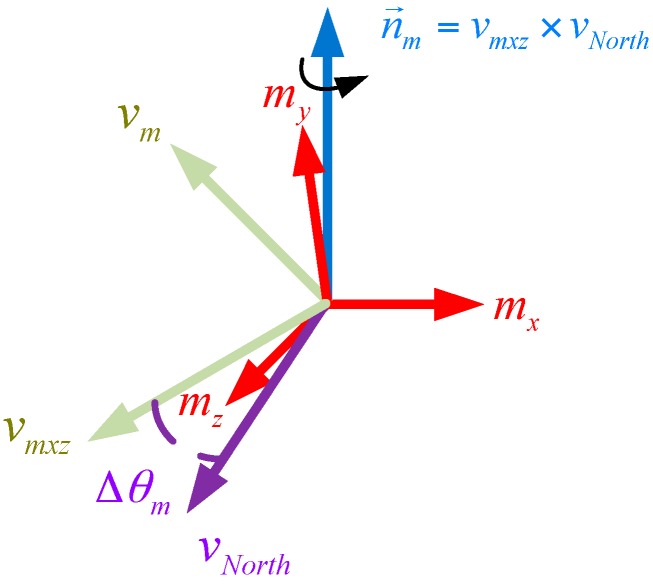
Correcting the estimated direction of the magnetic field using magnetometer.

If the yaw in quaternion $q_{a}$ is accurate, $v_{mxz}$ will point northward. However, due to the probable magnetic distortion in the local magnetic field, there will be a deviation between $v_{mxz}$ and the reference vector pointing northward $v_{North}=\left[\begin{array}{ccc} 1 & 0 & 0 \end{array}\right]$. As shown in Figure 4, the correction for the deviation can be performed by rotating the estimated orientation $q_{a}$ in (21) by the angle $\Delta \theta_{m}$ around the axis ${\vec{n}}_{m}$. Thus, the direction of $v_{mxz}$ will point northward as expected. The corresponding error quaternion $q_{me}$ and the estimated orientation $q_{m}$ can be obtained by:

(24) $$q_{me}=\cos\left(\frac{\Delta \theta_{m}}{2}\right)+{\vec{n}}_{m}\sin\left(\frac{\Delta \theta_{m}}{2}\right)$$

(25) $$q_{m}=q_{me}⊗q_{a}$$

where ${\vec{n}}_{m}=v_{mxz}\times v_{North}$, $\Delta \theta_{m}=a\cos\left(v_{mxz}\cdot v_{North}\right)$.

It should be noted that the step two correction needs only conducted when the magnetic field intensity is stable, otherwise, it can be skipped. The external magnetic distortion can be detected by the following:

(26) $$magnetic\_distortion=\{\begin{array}{ll} T, & if\left|\left\|m_{k+1}^{b}\right\|-\left\|h\right\|\right|>x_{m}\\ F, & otherwise \end{array},k=0,1,2\ldots$$

where $\left\|m_{k+1}^{b}\right\|$ is the norm of the magnetic field measured from the tri-axis magnetometer at time step *k* + 1; and ‖h‖ is the local magnetic field norm and is supposed to be constant.

Finally, we can generalize the computed quaternion qk+1c as follows:

(27) qk+1c={qm⊗qk,if magnetic_distortion=Fqa⊗qk,otherwise,k=0,1,2…

where $q_{k}$ is the unit quaternion obtained from the last optimal estimate of the proposed Kalman filter.

#### 2.2.3. Kalman Filter Fusion

The computation of the proposed Kalman filter starts with the initial condition:

(28) $$\left\{ \begin{array}{l} {\hat{X}}_{0}=E\left[X_{0}\right]\\ P_{0}=E\left[\left(X_{0}-{\hat{X}}_{0}\right){\left(X_{0}-{\hat{X}}_{0}\right)}^{T}\right] \end{array}\right.$$

The initial estimate quaternion ${\hat{X}}_{0}$ is determined by (8)–(11) during alignment. The initial covariance matrix $P_{0}$ is always given a large positive value in order to achieve a stable filter and it is determined that $P_{0}=10\cdot I_{4\times 4}$. $I_{4\times 4}$ is the 4×4 identity matrix.

The next step is to project the state and covariance estimates from time step *k*−1 to step *k*:

(29) $$\left\{ \begin{array}{l} {\hat{X}}_{k+1}^{-}=\exp\left(\Omega_{k}\Delta T\right)X_{k}\\ P_{k+1}^{-}=\exp\left(\Omega_{k}\Delta T\right)P_{k}\exp{\left(\Omega_{k}\Delta T\right)}^{T}+Q_{k} \end{array}\right.$$

where $\exp\left(\Omega_{k}\Delta T\right)$ is the discrete time state transition matrix in (15) and (16); and $Q_{k}$ is the process noise covariance associated to the quaternion and can be given by (36).

Then, the Kalman gain is calculated as:

(30) $$K_{k+1}=P_{k+1}^{-}{\left(P_{k+1}^{-}+R_{k+1}\right)}^{-1}$$

where $R_{k+1}$ is the measurement noise covariance and is determined by (41).

The final step is to obtain the posterior error covariance estimate:

(31) $$\left\{ \begin{array}{l} {\hat{X}}_{k+1}={\hat{X}}_{k+1}^{-}+K_{k+1}\left[Z_{k+1}-{\hat{X}}_{k+1}^{-}\right]\\ P_{k+1}=\left(I-K_{k+1}\right)P_{k+1}^{-} \end{array}\right.$$

where $Z_{k+1}$ is the computed quaternion given by (27).

From the process of the Kalman filter mentioned above, we can obtain the optimal estimated quaternion and finally calculate the 3-D attitude of the body.

### 2.3. Noise Characteristics

#### 2.3.1. Process Noise Covariance Determination

The covariance of process noise (quaternion) is mainly derived from the measurement of the gyroscope, and the noise of the gyroscope can influence the quaternion by the following equation:

(32) $$\left[\begin{array}{c} \dot{q}0\\ \dot{q}1\\ \dot{q}2\\ \dot{q}3 \end{array}\right]=\frac{1}{2}\left[\begin{array}{cccc} 0 & -\omega_{x} & -\omega_{y} & -\omega_{z}\\ \omega_{x} & 0 & \omega_{z} & -\omega_{y}\\ \omega_{y} & -\omega_{z} & 0 & \omega_{x}\\ \omega_{z} & \omega_{y} & -\omega_{x} & 0 \end{array}\right]\left[\begin{array}{c} q0\\ q1\\ q2\\ q3 \end{array}\right]$$

Assuming that the measurement of gyroscope $\omega ={\left[\begin{array}{ccc} \omega_{x} & \omega_{y} & \omega_{z} \end{array}\right]}^{T}$ consists of two components: the ideal value $\bar{\omega }={\left[\begin{array}{ccc} {\bar{\omega }}_{x} & {\bar{\omega }}_{y} & {\bar{\omega }}_{z} \end{array}\right]}^{T}$ and the drift of the gyroscope in the body frame $\delta \omega ={\left[\begin{array}{ccc} \delta \omega_{x} & \delta \omega_{y} & \delta \omega_{z} \end{array}\right]}^{T}$, that is: $\omega =\bar{\omega }+\delta \omega$. Then, the state equation can be rewritten as:

(33) $$\left[\begin{array}{c} \dot{q}0\\ \dot{q}1\\ \dot{q}2\\ \dot{q}3 \end{array}\right]=\frac{1}{2}\left[\begin{array}{cccc} 0 & -{\bar{\omega }}_{x} & -{\bar{\omega }}_{y} & -{\bar{\omega }}_{z}\\ {\bar{\omega }}_{x} & 0 & {\bar{\omega }}_{z} & -{\bar{\omega }}_{y}\\ {\bar{\omega }}_{y} & -{\bar{\omega }}_{z} & 0 & {\bar{\omega }}_{x}\\ {\bar{\omega }}_{z} & {\bar{\omega }}_{y} & -{\bar{\omega }}_{x} & 0 \end{array}\right]\left[\begin{array}{c} q0\\ q1\\ q2\\ q3 \end{array}\right]+\frac{1}{2}\left[\begin{array}{ccc} q1 & q2 & q3\\ -q0 & q3 & -q2\\ -q3 & -q0 & q1\\ q2 & -q1 & -q0 \end{array}\right]\left[\begin{array}{c} \delta \omega_{x}\\ \delta \omega_{y}\\ \delta \omega_{z} \end{array}\right]$$

We can separate the process noise w from the above equation as shown in:

(34) $$w=\frac{1}{2}\left[\begin{array}{ccc} q1 & q2 & q3\\ -q0 & q3 & -q2\\ -q3 & -q0 & q1\\ q2 & -q1 & -q0 \end{array}\right]\left[\begin{array}{c} \delta w_{x}\\ \delta w_{y}\\ \delta w_{z} \end{array}\right]$$

In the discrete time system, the process noise can be expressed as:

(35) $$w_{k}=\frac{\Delta T}{2}G_{k}v_{gk}=\frac{\Delta T}{2}\left[\begin{array}{ccc} q1 & q2 & q3\\ -q0 & q3 & -q2\\ -q3 & -q0 & q1\\ q2 & -q1 & -q0 \end{array}\right]v_{gk}$$

where ΔT is the sample time (we set 0.001 s in our implementation); and $v_{gk}$ is the mutually uncorrelated zero-mean white Gaussian noise with covariance matrix $\sum_{g}=\delta^{2}{Ι}_{3\times 3}$. Then, the process noise covariance matrix $Q_{k}$ is presented in the following:

(36) $$Q_{k}=E\left(w_{k}w_{k}{}^{T}\right)=\frac{\Delta T^{2}}{4}G_{k}\sum_{g}G_{k}{}^{T}$$

#### 2.3.2. Measurement Noise Covariance Determination

Let us first define the notion that will be used in the following section. We can define the measurement vector $u={\left[\begin{array}{cccccc} a_{x} & a_{y} & a_{z} & m_{x} & m_{y} & m_{z} \end{array}\right]}^{T}$, the measurement noise covariance matrix of the accelerometer and magnetometer $\Sigma_{u}=\left[\begin{array}{cc} \Sigma_{acc} & 0\\ 0 & \Sigma_{mag} \end{array}\right]$, the local gravity field norm $\left\|a\right\|=9.7997m/s^{2}$ and the local magnetic field norm ‖h‖=53μT.

According to the standard deviation obtained from the measurement of the accelerometer, we could easily construct the covariance matrix when the measurement vector is normalized:

(37) $$\sum_{acc}=\frac{1}{{\left\|a\right\|}^{2}}\left[\begin{array}{ccc} {\sigma_{ax}}^{2} & 0 & 0\\ 0 & {\sigma_{ay}}^{2} & 0\\ 0 & 0 & {\sigma_{az}}^{2} \end{array}\right]$$

Similarity, for the normalized magnetic field vector, the covariance matrix can be written as follows:

(38) $$\sum_{mag}=\frac{1}{{\left\|h\right\|}^{2}}\left[\begin{array}{ccc} {\sigma_{mx}}^{2} & 0 & 0\\ 0 & {\sigma_{my}}^{2} & 0\\ 0 & 0 & {\sigma_{mz}}^{2} \end{array}\right]$$

From the relationship between the observation vector in the body frame and the navigation frame in Equation (18), we can conclude that the measurement of accelerometer and magnetometer u is a function of $q={\left[\begin{array}{cccc} q0 & q1 & q2 & q3 \end{array}\right]}^{T}$, that is:

(39) $$u=\left[\begin{array}{c} a_{x}\\ a_{y}\\ a_{z}\\ m_{x}\\ m_{y}\\ m_{z} \end{array}\right]=\left[\begin{array}{c} 2q0q3+2q1q2\\ q0^{2}-q1^{2}+q2^{2}-q3^{2}\\ 2q2q3+2q0q1\\ \left(q0^{2}+q1^{2}-q2^{2}-q3^{2}\right)m_{N}+\left(2q0q3+2q1q2\right)m_{U}\\ \left(-2q0q3+2q1q2\right)m_{N}+\left(q0^{2}-q1^{2}+q2^{2}-q3^{2}\right)m_{U}\\ \left(2q0q2+2q1q3\right)m_{N}+\left(-2q0q1+2q2q3\right)m_{U} \end{array}\right]$$

and we can rewrite the function as [32]:

(40) q=f(u)

It is clear that q is a nonlinear function of u; we can linearize it by first-order Taylor expansion around the current estimate using the Jacobian matrix as follows:

(41) q=Ju

(42) $$J=\frac{\partial q}{\partial u}=\left[\begin{array}{cccccc} \frac{\partial q_{0}}{\partial a_{x}} & \frac{\partial q_{0}}{\partial a_{y}} & \frac{\partial q_{0}}{\partial a_{z}} & \frac{\partial q_{0}}{\partial m_{x}} & \frac{\partial q_{0}}{\partial m_{y}} & \frac{\partial q_{0}}{\partial m_{z}}\\ \frac{\partial q_{1}}{\partial a_{x}} & \frac{\partial q_{1}}{\partial a_{y}} & \frac{\partial q_{1}}{\partial a_{z}} & \frac{\partial q_{1}}{\partial m_{x}} & \frac{\partial q_{1}}{\partial m_{y}} & \frac{\partial q_{1}}{\partial m_{z}}\\ \frac{\partial q_{2}}{\partial a_{x}} & \frac{\partial q_{2}}{\partial a_{y}} & \frac{\partial q_{2}}{\partial a_{z}} & \frac{\partial q_{2}}{\partial m_{x}} & \frac{\partial q_{2}}{\partial m_{y}} & \frac{\partial q_{2}}{\partial m_{z}}\\ \frac{\partial q_{3}}{\partial a_{x}} & \frac{\partial q_{3}}{\partial a_{y}} & \frac{\partial q_{3}}{\partial a_{z}} & \frac{\partial q_{3}}{\partial m_{x}} & \frac{\partial q_{3}}{\partial m_{y}} & \frac{\partial q_{3}}{\partial m_{z}} \end{array}\right]$$

where J is the 4×6 Jacobian matrix of the quaternion q, and the corresponding covariance matrix of q can be calculated as:

(43) $$\sum_{q}=J\sum_{u}J^{T}$$

### 2.4. Hardware Design

**Figure 5:**
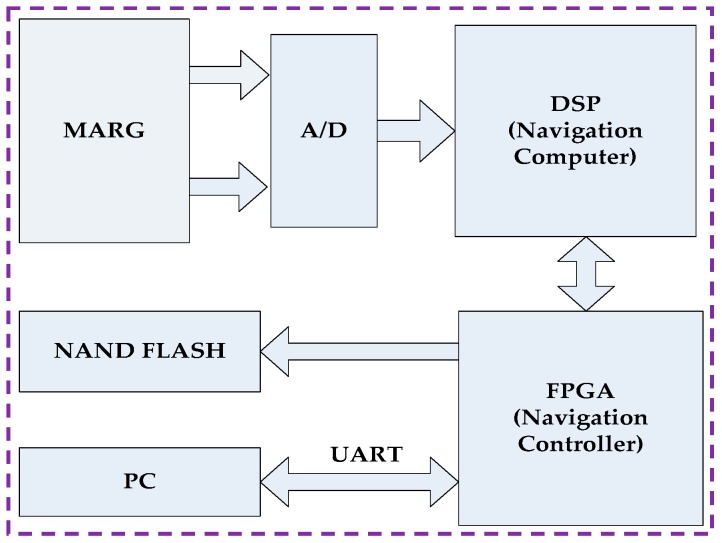
Structural diagram of the AHRS.

**Figure 6:**
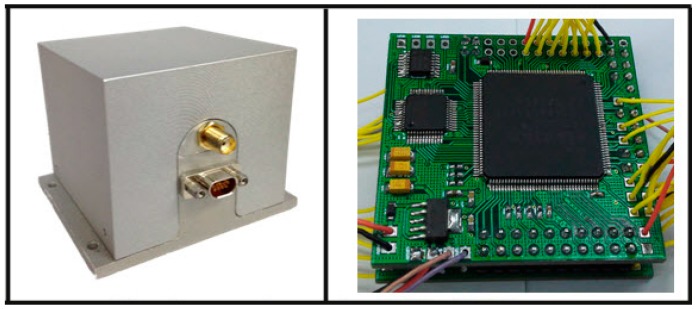
Homemade prototype of the AHRS. Homemade prototype of the AHRS (**Left**) and its circuit component (**Right**).

The proposed method was tested on an AHRS produced by us. The system was equipped with a three single-axis CRM100 MEMS gyros, three single-axis MS9000 MEMS accelerometers and a tri-axis HMC1043L AMR magnetometer. The full-scale range of the accelerometer, gyroscopes and magnetometer were ±2 g, ±300 °/s and ±6 gauss, respectively. They all provide analog outputs, so two six-channel 16-bit analog-to-digital converters (ADC) ADS8365 were used to acquire the raw data. Additionally, in order to improve the computational efficiency and storage speed, the hardware structure based on FPGA (field programmable gate array) + DSP (digital signal processor) was selected. FPGA is used to gather raw data from sensors and then transmit the estimated attitude to PC (personal computer) or Flash, while DSP is used for data fusion and orientation computation for its excellent performance. The data collection was performed through an RS-232 (recommended standard) communication serial port at 115,200 baud rate for experiment analysis. Overall, the dimensions of the AHRS were approximately 60 mm × 60 mm × 60 mm. A structural diagram of the hardware design is shown in Figure 5. Figure 6 shows a picture of the proposed AHRS.

### 2.5. Experimental Setup

To verify the proposed method, three kinds of experiments were carried out by the advanced navigation system research group of the North University of China (Taiyuan, China). The first experiment was implemented on a tri-axis turntable for static and slow-movement performance testing. The second test used a ground vehicle equipped with a high precision attitude reference system to validate the robustness of the attitude estimation in fast-movement situations. The third experiment was to test the static accuracy when the proposed AHRS is subjected to magnetic field variations. The bias and random error standard deviation of the MARG sensors is shown in Table 1. These values are used to calculate the process noise and measurement noise covariance matrices in the Kalman filter. The parameters of the proposed Kalman filter adopted in the experiments are as follows:

Q_k_ = diag (1e^−6^, 1e^−6^, 1e^−6^, 1e^−6^)

R_k_ = diag (0.0015, 0.0015, 0.0015, 0.0015)

where diag (,) represents a diagonal matrix.

A. Experiment 1 (Static and Low Movement Test)

**Figure 7:**
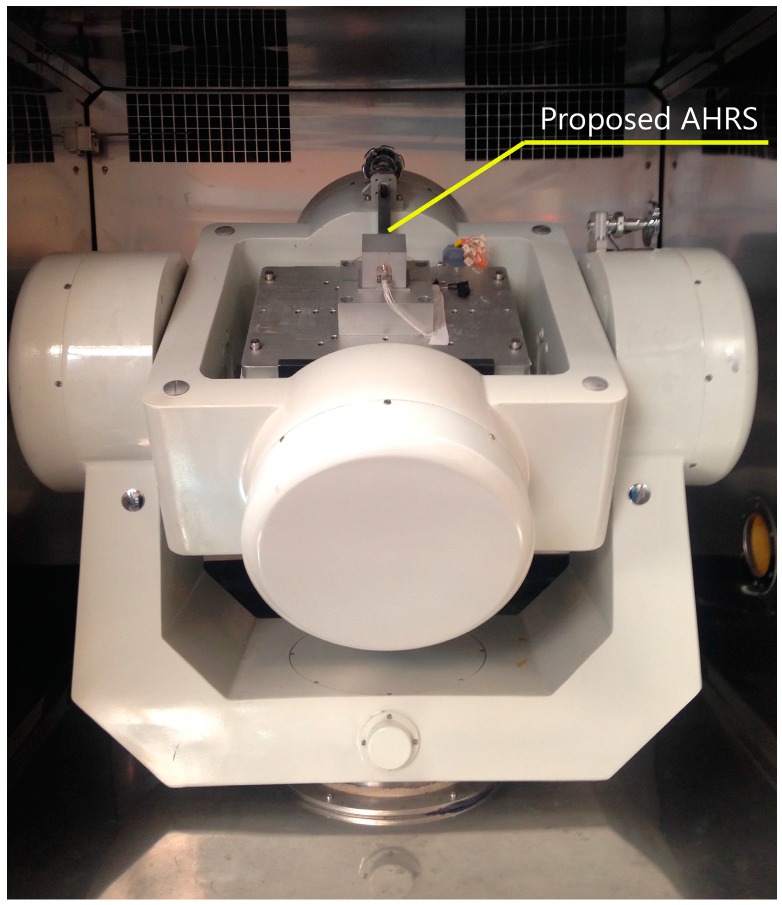
Physical map of three-axis turntable testing.

The static and low-movement performance of the proposed AHRS was evaluated by using a high-precision tri-axis turntable as shown in Figure 7. Since the turntable is able to obtain a position accuracy of 3” at a rate range from 0 to 400 °/s, its motion feedback can be regarded as the true reference. Considering that the turntable is ferrous material and the magnetometer is influenced by it, we used the accuracy of pitch and roll to represent the performance of the AHRS in the turntable test. In this experiment, the AHRS was initially fixed on the table with its *x*-*y*-*z* axis aligned with the front-upper-right. In order to test the static performance of the AHRS, the system was rotated 10° about the z axis and then kept static for 10 s every cycle. The test angle was between −45° and 45°. In the slow movement test, the motion of the system was set according to a sine wave with an amplitude of 20° and a frequency of 0.5 Hz. The test time was 100 s.

B. Experiment 2 (Fast Movement Test)

**Figure 8:**
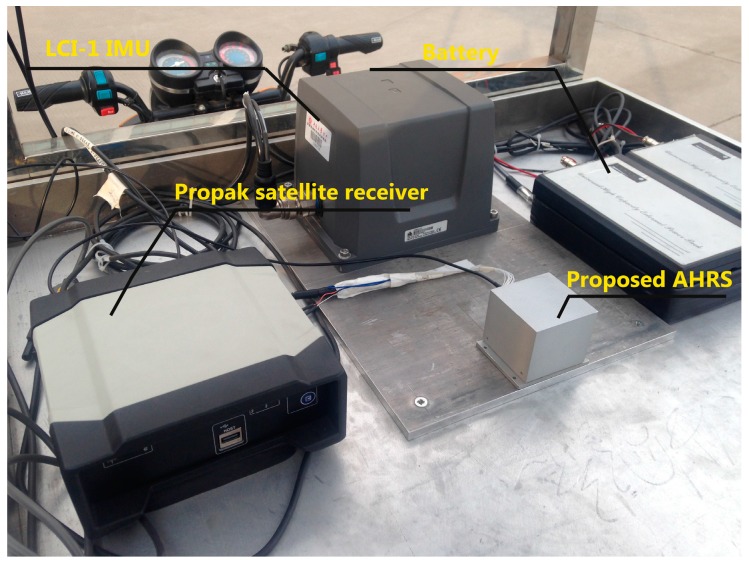
The vehicle test platform and designed AHRS used in this experiment.

**Figure 9:**
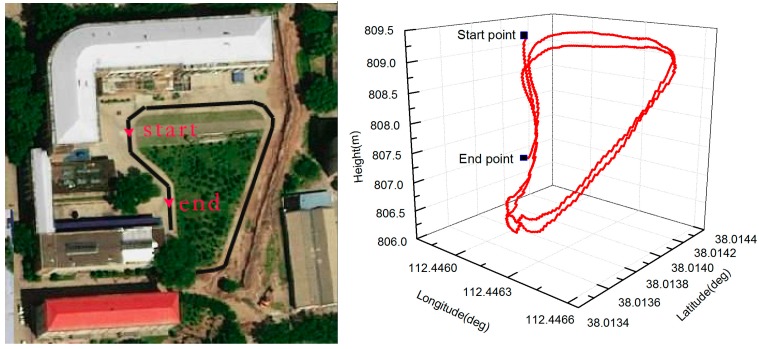
The reference trajectory of the vehicle. The two-dimensional reference trajectory of the vehicle (**Left**) and the corresponding three-dimensional trajectory (**Right**).

To evaluate the performance of the proposed algorithm in a fast movement application, we mounted the designed AHRS on a land vehicle platform as shown in Figure 8. The test vehicle platform consisted of an LCI-1 tactical grade IMU (inertial measurement unite) (whose specifications are depicted in Table 2) and a Propak satellite receiver, which uses Novatel SPAN (synchronized position attitude navigation) as the reference solution. The accuracy of the reference solution in these conditions is summarized in Table 3. The reference trajectory of the vehicle in this field test is shown in Figure 9.

C. Experiment 3 (Magnetic Distortion Test)

For the experiment with magnetic distortion, the proposed AHRS was mounted on a level platform and kept static during the whole test. The *x*-*y*-*z* axes of the system were aligned with the N-U-E (North-Up-East) directions, respectively. After a small period of initialization, we provided the magnetic disturbances by approaching an iron-made stick to the system for about 5 s and then we removed it. The output of the Novatel SPAN system was employed as the reference, and the update frequency of reference was 50 Hz.

## 3. Results and Discussion 

### 3.1. Tri-Axis Turntable Experiments for the Proposed AHRS

**Figure 10:**
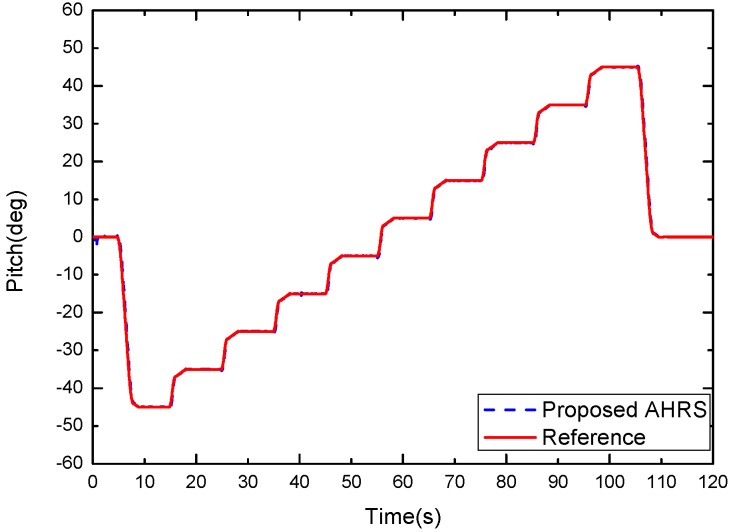
Pitch estimate produced by the proposed Kalman filter (blue dash curve, $\mu_{a}=0.9$) and the turntable reference(red solid curve).

**Figure 11:**
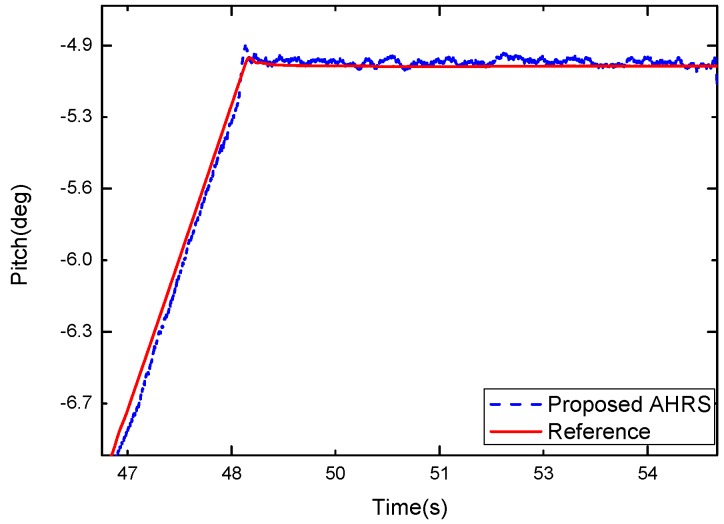
Zoomed-in view of the pitch estimate (dash curve) from the Kalman filter and the turntable reference (solid curve).

**Figure 12:**
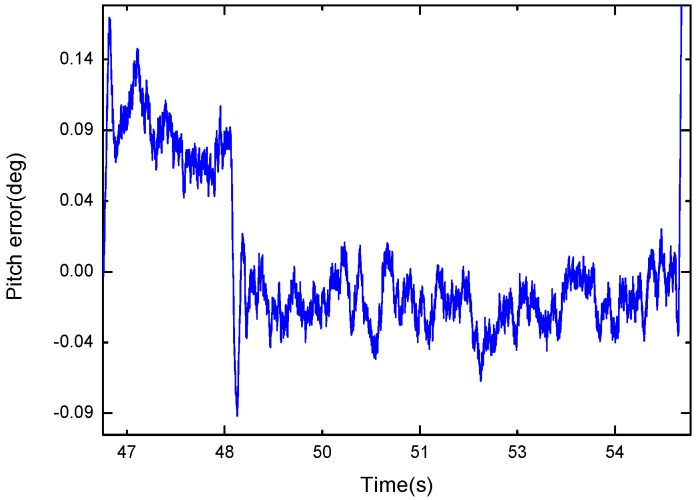
Difference between the pitch estimate and turntable reference.

Figure 10 shows the results of the experiment in the static condition. Figure 11 is replotted in a zoomed-in view for the time period of 47–55 s. The blue dash curve represents the orientation estimated by the proposed algorithm, and the red solid curve is the turntable reference. The difference between the two curves is shown in Figure 12. It can be observed that the proposed algorithm with $\mu_{a}=0.9$ was able to estimate the pitch angle correctly in the static state for the time periods 49–55 s. During the rotation motion for the time periods 47–49 s, a relatively large error was produced. This is because that in this case the accelerometer had a relative large weight and it cannot distinguish the gravity from the external acceleration, thus the filter is not able to estimate the direction of the gravity correctly.

**Figure 13:**
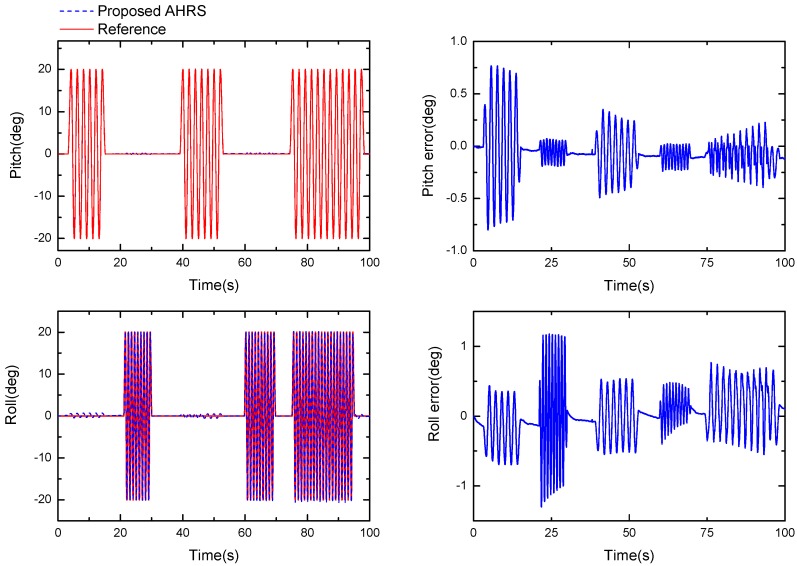
Orientation estimate produced by the proposed algorithm (**left**, $\mu_{a}=0.005$) and the difference between the estimate and reference (**right**).

During the slow movement test, we first executed the sine sway of the AHRS around its *z*-axis, then, we repeated the same maneuvers around the *x*-axis. After repeating this twice, the sway was conducted around the *z*-axis and *x*-axis simultaneously. No sway around the *y*-axis was performed since the experiment was conducted in a magnetically nonhomogeneous environment and the output of sways around the *y*-axis would be affected by the magnetic distortion. Figure 13 shows the performance of the proposed Kalman filter in this condition. The graph to the left shows the pitch angle and roll angle estimated by the proposed algorithm, and the graph to the right shows the difference between the estimated and the real value. It can be seen that the slow movement accuracy of the filter is better than 0.5°, and the maximum error is about 1°.

### 3.2. Experiments on the Driving Vehicle 

**Figure 14:**
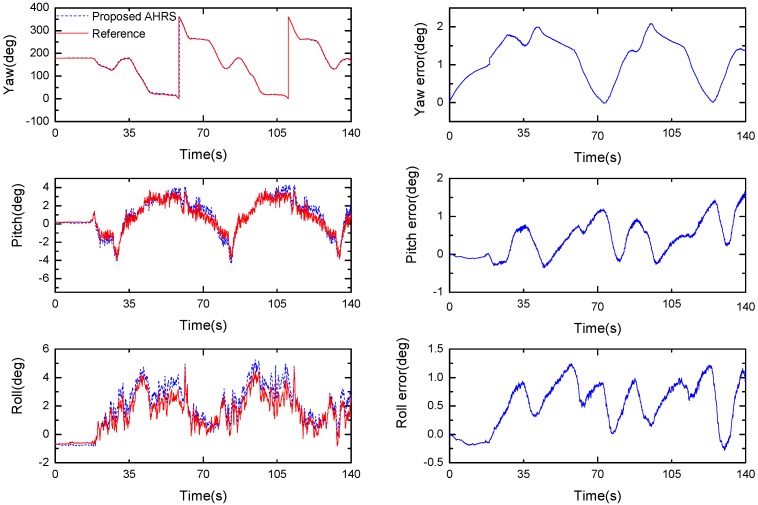
Orientation estimate produced by the proposed algorithm (**left**, $\mu_{a}=0.001$) and the difference between the estimate and reference (**right**).

Figure 14 shows the performance of the proposed Kalman filter under fast movement conditions. It can be seen that the orientation of the vehicle can be effectively tracked throughout the duration of the whole experiment. The root mean square error (RMSE) of the pitch and roll angle are less than 0.8°, and approximately 1° for the heading angle during the whole fast movement test.

For better evaluation of the proposed KF, the results are compared with other popular algorithms, including the conventional EKF with 7-D state vector (4-D quaternion and 3-D gyro bias) and Madgwick’s complementary filter. To quantitatively describe the estimation performance of the three algorithms, the RMSE of the attitude estimation corresponding to each algorithm under different working conditions are listed in Table 4. It can be seen that the three algorithms achieved similar levels of performance under static or slow movement condition. When the AHRS maneuvered in fast movement situation, it is obvious that the orientation estimate accuracy of Madgwick’s complementary filter significantly degrades. This is because Madgwick’s gradient descent algorithm is a constant gain filter, and a fixed-step size gradient descent cannot track the dynamic characteristics of the vehicle adaptively. From Table 4, we can also see that the RMSE of the proposed KF is slightly lower than that of the EKF. It is demonstrated that our proposed KF has better performance than that of the two algorithms in attitude estimation under dynamic conditions. 

### 3.3. Experiments with Magnetic Distortion

**Figure 15:**
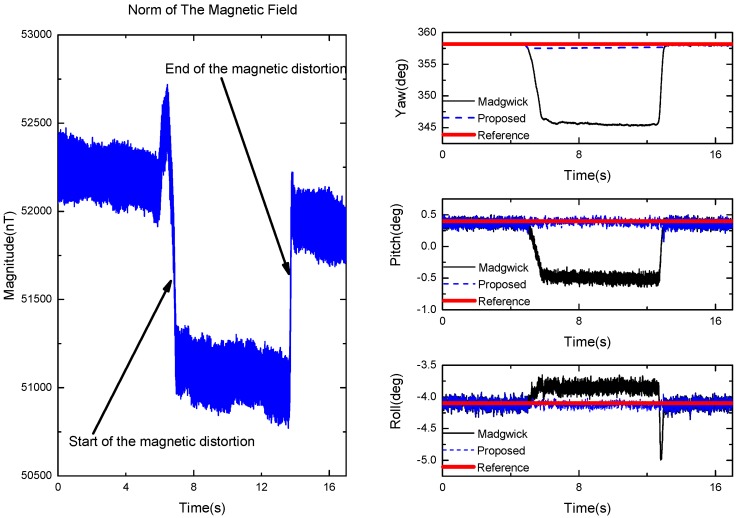
The norm of the magnetic field (**left**) and the orientation produced by the proposed algorithm (blue, $\mu_{a}=0.9$), Madgwick’s complementary filer (black, β=0.8 ) (**right**).

The norm of the magnetometer’s measurements is shown in Figure 15 (left). It can be observed that in the absence of magnetic distortion, the local magnetic field strength is 52 uT. However, due to the effect of the induced magnetic disturbance, the norm of measurements was evidently changed. The performance of the proposed Kalman filter in orientation estimation under a magnetic disturbance environment is shown in Figure 15 (right). The solid curve represents the attitude estimated from the proposed Kalman filter, and the dash curve is the orientation estimated from Madgwick’s gradient descent algorithm. It can be seen that the proposed algorithm can detect the magnetic disturbance intelligently and is effectively immune to the external magnetic disturbance. On the other hand, the pitch/roll from Madgwick’s gradient descent algorithm are easily affected when we approach the magnet to the AHRS, and this is converted back to the right value when the magnetic disturbance is removed. The main reason for this is that the two-step geometrically intuitive correction (TGIC) approach used in our KF has the ability to decouple the correction of the pitch/roll from the correction of the yaw. From Figure 15 (right), we can also see that the estimation of the roll from Madgwick’s filter will experience a relevant error when the magnet is removed. It means that the pitch/roll from Madgwick’s filter are easily affected by variable fluxes.

### 3.4. Time Consumption Evaluation

In this section, we would like to validate the time consumption performance of the proposed filter along with the EKF (four quaternion components as the state vector) and Madgwick’s filter. The three algorithms were profiled in MATLAB by using the raw data logged from our homemade AHRS at 1 KHz. The Matlab program was executed on an Intel Core(TM) i3-4160 3.6 GHz processor. Table 5 shows the results of the average execution time of each calculation cycle. By comparing the average time consumption, we can see that Madgwick’s filter consumes the least and is the fastest. This is because that the constant gain in Madgwick’s filter is a simpler estimation method without statistical analysis and a mass matrix operation. In addition, we can see that the proposed method is faster than the standard EKF, which is due to the fact that the observation quaternion produced by the TGIC avoids the linearization error of the measurement equations and the computation of the Jacobian matrix required in standard EKF. Thus, by comparing the estimation accuracy and the computational time of the three algorithms comprehensively, we can conclude that the proposed filter reaches a tradeoff between time consumption and accuracy, making it more suitable in low-configuration embedded applications. 

## 4. Conclusions

In this paper, a novel two-layer Kalman filter was proposed for the orientation estimation of AHRS by fusing data from MARG sensors. The Kalman filter was significantly simplified by preprocessing the accelerometer and magnetometer information using a two-step geometrically-intuitive correction (TGIC) approach. Compared with the traditional external quaternion estimation algorithm, the use of two-step correction decouples the accelerometer and magnetometer information. This decoupling eliminates the influence of magnetic interference on the current estimation of pitch/roll. In addition, the quaternion produced by the TGIC is utilized as the input observation vector for the Kalman filter, which avoids the linearization error of measurement equations and reduces the computational complexity of the filter. By carrying out several experiments, the performance of the proposed filter in static, dynamic and magnetic distortion conditions was verified. The experimental results indicate that the proposed Kalman filter is able to provide relatively faster and more accurate real-time orientation information in different working conditions.

## Figures and Tables

**Table 1:** Specifications of the sensors in the proposed AHRS.

	Bias	Standard Deviation
**Gyroscope**	[−6.7e^−3^ −4.9e^−3^ −9.7e^−3^] (rad/s)	[0.001 0.001 0.001] (rad/s)
**Accelerometer**	[0.09 −0.01 0.7] (m/s^2^)	[0.039 0.036 0.039] (m/s^2^)
**Magnetometer**	[280 −439 −22] (mGauss)	[0.22 1.11 0.28] (mGauss)

**Table 2:** The characteristics of LCI-1 IMU.

	Gyroscope	Accelerometer
**Range**	±800 °/s	±40 g
**Bias**	<1°/h	<1 mg
**Scale Factor**	<500 ppm	<1000 ppm

**Table 3:** System performance of the reference system.

Position(CEP)		Attitude (1σ Value)		
		Yaw	Pitch	Roll
Accuracy	0.3–5 m	<0.01°	<0.01°	<0.01°

**Table 4:** RMS (root mean square) errors of the proposed AHRS in various working conditions.

Case	Filters	Yaw/°	Pitch/°	Roll/°
**Static**	Madgwick	—	0.0362	0.0375
	EKF	—	0.0384	0.0331
	Proposed KF	—	0.0252	0.0341
**Slow Movement**	Madgwick	—	0.3543	0.4350
	EKF	—	0.2088	0.3345
	Proposed KF	—	0.2159	0.3614
**Fast Movement**	Madgwick	2.0327	1.0268	1.1563
	EKF	1.5365	0.7253	0.6525
	Proposed KF	1.2765	0.6276	0.6686

**Table 5:** Computational time of the proposed KF, Madgwick’s filter and EKF.

Algorithm	Mean Time Consumption (ms)	Standard deviation (ms)
Proposed KF	0.1832	0.0191
Madgwick’s filter	0.1255	0.0238
EKF	0.2028	0.0360
