# Correction: A New Quaternion-Based Kalman Filter for Real-Time Attitude Estimation Using the Two-Step Geometrically-Intuitive Correction Algorithm. *Sensors* 2017, *17*, 2146

**DOI:** 10.3390/s17112530

**Published:** 2017-11-03

**Authors:** Kaiqiang Feng, Jie Li, Xiaoming Zhang, Chong Shen, Yu Bi, Tao Zheng, Jun Liu

**Affiliations:** 1 Key Laboratory of instrumentation Science & Dynamic Measurement, Ministry of Education, North University of China, Taiyuan 030051, China; b1506011@st.nuc.edu.cn (K.F.); zxm_auto@nuc.edu.cn (X.Z.); shenchong@nuc.edu.cn (C.S.); b1506009@st.nuc.edu.cn (Y.B.); s1506044@st.nuc.edu.cn (T.Z.); liuj@nuc.edu.cn (J.L.); 2 National Key Laboratory for Electronic Measurement Technology, North University of China, Taiyuan 030051, China

The authors wish to make the following corrections to their paper [1]:

1. In page 7, “The two-step correction process is described as follows:” should be revised as “The two-step correction process is described as follows [31]:”. Meanwhile, the corresponding reference “31. Del Rosario, M.B.; Lovell, N.H.; Redmond, S.J. Quaternion-based complementary filter for attitude determination of a smartphone. *IEEE Sens. J.* **2016**, *16*, 6008–6017.” should be added in the references part.
2. In page 11, “and we can rewrite the function as:” should be revised as “and we can rewrite the function as [32]:”. Meanwhile, the corresponding reference “32. Valenti, R.G.; Dryanovski, I.; Xiao, J.Z. A linear kalman filter for marg orientation estimation using the algebraic quaternion algorithm. *IEEE Trans. Instrum. Meas.* **2016**, *65*, 467–481.” should be added in the references part.

The authors would like to apologize for any inconvenience caused to the readers by these changes.
